# Ancient and Contemporary DNA Reveal a Pre-Human Decline but No Population Bottleneck Associated with Recent Human Persecution in the Kea (*Nestor notabilis*)

**DOI:** 10.1371/journal.pone.0118522

**Published:** 2015-02-26

**Authors:** Nicolas Dussex, Nicolas J. Rawlence, Bruce C. Robertson

**Affiliations:** Allan Wilson Centre for Molecular Ecology and Evolution, Department of Zoology, University of Otago, PO Box 56, Dunedin 9054, New Zealand; University of York, UNITED KINGDOM

## Abstract

The impact of population bottlenecks is an important factor to consider when assessing species survival. Population declines can considerably limit the evolutionary potential of species and make them more susceptible to stochastic events. New Zealand has a well documented history of decline of endemic avifauna related to human colonization. Here, we investigate the genetic effects of a recent population decline in the endangered kea (*Nestor notabilis*). Kea have undergone a long-lasting persecution between the late 1800s to 1970s where an estimated 150,000 kea were culled under a governmental bounty scheme. Kea now number 1,000–5,000 individuals in the wild and it is likely that the recent population decline may have reduced the genetic diversity of the species. Comparison of contemporary (n = 410), historical (n = 15) and fossil samples (n = 4) showed a loss of mitochondrial diversity since the end of the last glaciation (Otiran Glacial) but no loss of overall genetic diversity associated with the cull. Microsatellite data indicated a recent bottleneck for only one population and a range-wide decline in *N_e_* dating back some 300 – 6,000 years ago, a period predating European arrival in NZ. These results suggest that despite a recent human persecution, kea might have experienced a large population decline before stabilizing in numbers prior to human settlement of New Zealand in response to Holocene changes in habitat distribution. Our study therefore highlights the need to understand the respective effects of climate change and human activities on endangered species dynamics when proposing conservation guidelines.

## Introduction

Genetic diversity is an essential component of both the short and long-term persistence of species. It can be regarded as the basis for evolutionary potential [1] and favors an adaptive response to changing environments [2]. Endangered species with small population sizes often show low genetic diversity [3, 4], which compromises long-term viability and adaptability, and increases chances of inbreeding [4–6]. Even though recent population declines or extinctions are often attributed to human activities (e.g. habitat destruction, over-exploitation) [7–9], humans are not the sole driver of population dynamics of species. For instance, the population dynamics of the musk ox (*Ovibos moschatus*) were best explained by climate and associated habitat change [10] while the extinction of woolly mammoths (*Mammuthus primigenius*) appears to have been caused by the concomitant effect of over-hunting and climate change [11]. It is therefore necessary to identify the underlying cause of decline, to determine the appropriate management strategy of a species.

In order to distinguish anthropogenic from natural drivers of population decline, one can refer to pre-human fossil records of the distribution of species. However, such data is often lacking or incomplete, leading to a potential bias in the estimation of population size and oversimplification of population dynamics, as critically addressed by Rawlence et al. [7] and Allentoft et al. [12].

DNA-based approaches using contemporary samples only are routinely used to detect population declines [13–16] but can also have limited resolution. Indeed, a direct comparison of historical and contemporary genetic diversity is often advantageous because it allows the event associated with population fluctuations to be dated with more accuracy and provides a unique opportunity to study genetic variability in pre-bottleneck populations [17–19].

The avifauna of New Zealand evolved in the absence of mammalian predators [20, 21] and multiple lines of evidence support a history of population bottlenecks and extinctions linked to human colonization [8, 22]. The main cause of decline or population extinction is often attributed to introduced predators [23–26] or over-hunting [7–8, 27]. Additionally, some species were historically persecuted. For instance, the kea (*Nestor notabilis*), an endemic alpine parrot prehistorically present in both the North and South Islands of New Zealand [28–32] and now only found in the South Island was considered a pest [33]. Its inquisitiveness [34, 35], opportunistic feeding behavior [36–38] and more notably attacks on sheep [39–41] prompted a bounty scheme where over 150,000 kea were killed between the late 1800s and 1971 (when they received partial legislative protection). Today the species is mainly found in the alpine areas of the South Island above the tree line [42] while a few populations remain in podocarp forest in Westland [43] but it is still declining due to ongoing predation by introduced mammals [25]. However, accurate estimates of current effective population size are difficult to obtain because kea are often found in remote and rugged habitat. The most commonly accepted and conservative estimates of contemporary population size range between 1,000–5,000 individuals [44]. The estimates of the number of birds culled and current population size therefore suggest that kea went through a significant population bottleneck since European arrival.

Previous results have shown that despite its strong flying capabilities, the kea is subdivided into three distinct genetic clusters and that this population structure is most likely the result of postglacial recolonization processes but not a recent human-induced fragmentation [45]. Kea populations might also have fluctuated through time in response to climate variations during the Pleistocene and Holocene as has been shown in many other New Zealand species [7, 46]. The history of kea thus provides an excellent opportunity to test for genetic signatures of population size changes associated with past climatic events and recent human activities.

Here we test whether kea have experienced pre-human population declines or if the species started to decline only recently because of human persecution. We first assess changes in genetic diversity over time using microsatellite and mitochondrial data comparing contemporary, historical and fossil samples. Second, we look for a signature of ancient and recent bottlenecks using conventional methods for microsatellite data. We also look for a signature of population decline using coalescent-based Bayesian methods and estimate its timing and magnitude. Due to the magnitude of the reported recent population collapse (i.e. loss of approximately 95% of the historical population), we expect to find extinct haplotypes and alleles among historical and fossil samples that are not present in contemporary samples and also a significant signature of a recent bottleneck.

## Material and Methods

### Ethics Statement

Fieldwork was conducted under the approval of the University of Otago Ethics Committee (No. 74/09) and DoC (Permits No. OT-26311; WC-26101-FAU). No permits were required to sample fossil material from museums. Permission to sample museum specimens was obtained by NJR from individual institutions.

### Sampling

**Fig 1:**
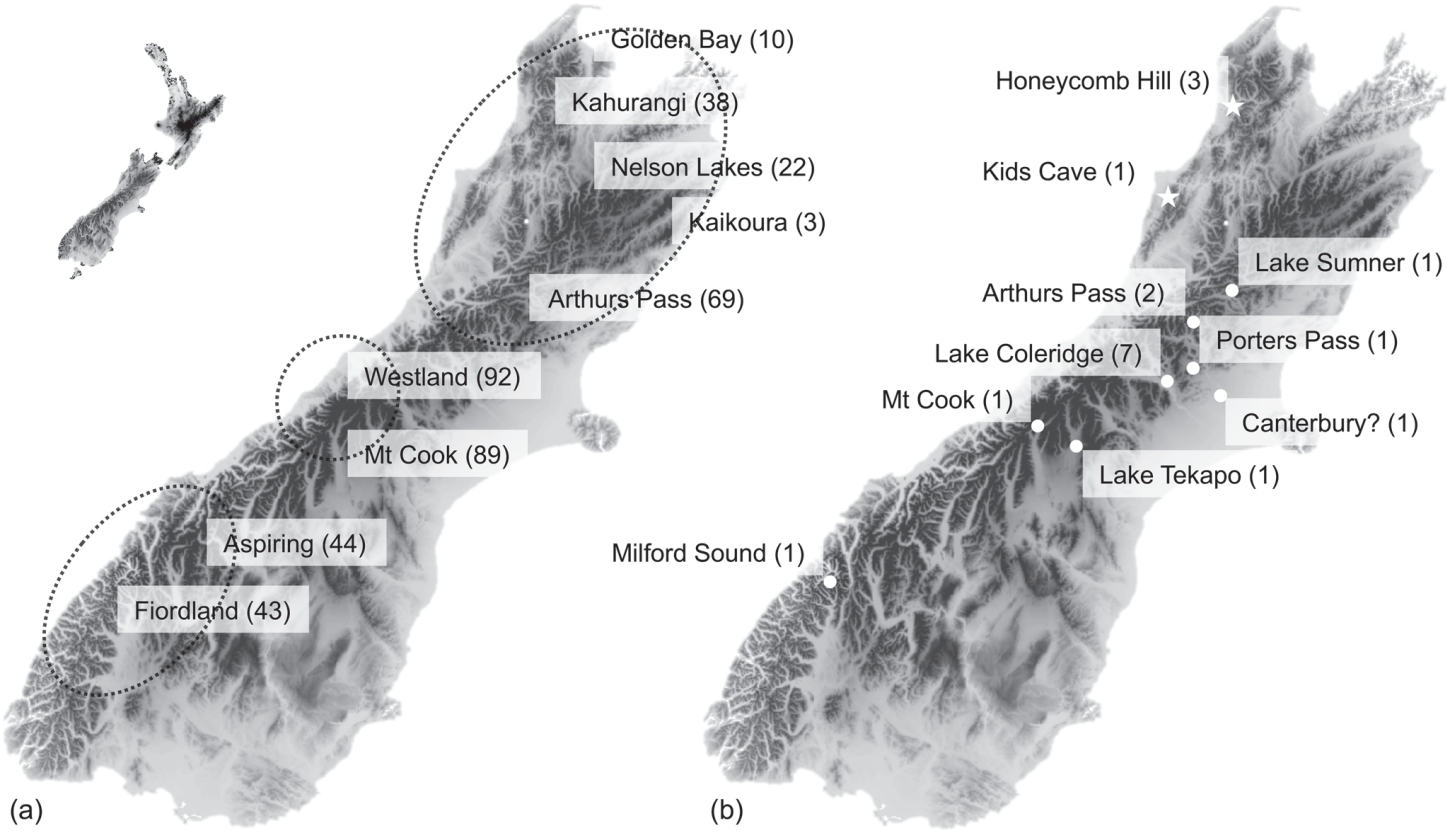
Sampling locations for (a) contemporary samples with circles representing genetic clusters in TESS as described in [45] and (b) the 15 historical skin samples (dots) and four fossil bones (stars) for kea. Numbers in brackets represent the sample size.

We utilized a contemporary dataset from birds caught between 2010 and 2012 comprising 91 mitochondrial (mtDNA) control region (CR) sequences and 410 nuclear microsatellite genotypes (17 microsatellite loci) as described in Dussex et al. [45]. Additionally, historical skins (n = 15) with robust locality data, and Late Glacial (10–14 kya) and Holocene fossil bones (< 11.6 kya; n = 4) [31, 47] from across the geographic range of kea were obtained from New Zealand and overseas museum collections (Table 1, Fig. 1). To ensure independence of samples, only common elements of the left or right orientation were sampled from an individual deposit, or bones were sampled from different stratigraphic units within a deposit (e.g. layers, squares). While most of the historical skins used in this study come from the Canterbury region, fossil samples from other regions extend the coverage to the whole contemporary range of the species.

**Table 1:** Historical and fossil samples used to obtain genotypic and sequencing data for the mitochondrial control region. Information on mitochondrial fragment length, sampling location and the corresponding cluster identified in TESS (See [45]) are given. The four fossil samples were not amplified for microsatellite markers. Abbreviations call on Hum, humerus; Tt, tibiotarsus.

**Sample number**	**Museum**	**Tissue type**	**Sample age/collection date**	**Region**	**Sampling location**	**mtDNA fragment length**	**Corresponding cluster**
NMNZ S.43574	National Museum of New Zealand Te Papa Tangarewa (NMNZ)	fossil bone	13–17 ky	West Coast	Kids Cave (0.5 cm depth; Hum)	450	North
NMNZ S.22664.1	(NMNZ)	fossil bone	11–14 ky	West Coast	Honeycomb Hill (Terrace edge, Graveyard; Ulna)	245	North
NMNZ S.22039.1	(NMNZ)	fossil bone	11–14 ky	West Coast	Honeycomb Hill (Terrace, Graveyard (L1+ L2 Ex2); Tt)	245	North
NMNZ S.22039.2	(NMNZ)	fossil bone	11–14 ky	West Coast	Honeycomb Hill (Terrace, Graveyard; Tt)	245	North
AlM LB2467	Auckland Museum (AlM)	toepad	1932	Canterbury	Lake Sumner	223*	North
AlM LB2466	(AlM)	toepad	1932	Canterbury	Canterbury	616	North
CM AV84	Canterbury Museum (CM)	toepad	1908	Canterbury	Wilberforce River (Lake Coleridge)	616	North
CM AV86	(CM)	toepad	1913	Canterbury	Wilberforce River (Lake Coleridge)	616	North
CM AV88	(CM)	toepad	1913	Canterbury	Lake Coleridge	223*	North
CM AV89	(CM)	toepad	1907	Canterbury	Windwhistle (Lake Coleridge)	616	North
CM AV90	(CM)	toepad	1913	Canterbury	Lake Coleridge	223*	North
CM AV91	(CM)	toepad	1913	Canterbury	Wilberforce River (Lake Coleridge)	616	North
NMW 12.208	Museum of Natural History Vienna, Austria (NMW)	toepad	1877	Canterbury	Arthur’s Pass	223*	North
NMW 12.209	(NMW)	toepad	1877	Canterbury	Arthur’s Pass	616	North
NMW 49.760	(NMW)	toepad	1877	Canterbury	Porter’s Pass	616	North
NMW 12.211	(NMW)	toepad	1877	Canterbury	Wilberforce River (Lake Coleridge)	616	North
AlM LB2469	(AlM)	toepad	1934	Fiordland	Milford Sound	616	South
CM AV2039	(CM)	toepad	1932	Mt Cook	Lake Tekapo, Canterbury Land District	616	Central
NHM 1927.12.18.125	Natural History Museum London (NHM)	toepad	1889	Mt Cook	Mt Cook	616	Central

*toepads with mtDNA fragment shorter than 450 bp were not used for comparison of contemporary and historical genetic diversity

### Ancient DNA extraction, amplification and sequencing

All ancient DNA (aDNA) extractions and PCR set up was carried out at the University of Otago in a purpose built aDNA laboratory physically isolated from other molecular laboratories [48]. Strict aDNA procedures were followed to minimize contamination of samples with exogenous DNA [49], including the use of negative extraction and PCR controls to ensure the authentication of sequences. DNA was extracted from historical skins using the Qiagen DNeasy Blood and Tissue Kit following the manufactures instructions, except the lysis step was performed as an overnight incubation at 56°C. Replicate extractions were performed on 10% of samples, chosen randomly. DNA was extracted from up to 250 mg of fossil bone powder following Rohland et al. [50]. PCR amplification and all downstream procedures were carried out in a modern genetics laboratory.

#### Microsatellite DNA amplification

Historical samples were genotyped for 17 microsatellite loci [45] using cycling conditions outlined in [51] with an increase in the number of cycles from 8 to 12 and from 25 to 30. All samples were independently amplified five times in order to decrease the chances of allelic dropout [52]. Microsatellite markers (> 200 bp) were not amplified for fossil samples, because of the short fragments obtained for mtDNA (∼150 bp) and associated low chance of successful amplification.

#### Mitochondrial DNA amplification

The mtDNA CR from historical skins was amplified and sequenced using four primer pairs (producing ∼250 bp fragments including primers) (S1 Table, S1 Fig.) and the same PCR conditions described in Dussex et al. [45] but with an increase in cycle number to 60.

Fossil samples were amplified and sequenced using seven primer pairs (∼150 bp fragments including primers) (S1 Table, S1 Fig.) to cover Domain I of the mtDNA CR. Each PCR reaction (20 µL) consisted of: 1 M Betaine (Sigma), 4 mM MgCl_2_ (Life Technologies), 1 x Gold Buffer II (Life Technologies), 2.5 mM dNTPs (Bioline), 250 nM each primer, 1.25 U of AmpliTaq Gold DNA Polymerase (Life Technologies), and 2 µL DNA. Unsuccessful PCRs were repeated with 2 U AmpliTaq Gold DNA Polymerase and 4 µL DNA or 2 µL 1:10 DNA. PCR thermocycling conditions consisted of 94°C 9 min, 60 cycles 94°C 30 s, 50°C 45 s, 72°C 1 min, followed by a final extension of 72°C 10 min.

PCR products were run on a 2% 1 x TAE agarose gel. PCR products were purified (using ExoSap (1.5 U ExoI, I U SAP; GE Healthcare) by incubation at 37°C for 30 min and 80°C for 15 min), and sequenced bi-directionally from independent PCR products using Big Dye Terminator technology and an ABI 3730xl. When an inconsistency between sequences from an individual was observed due to DNA damage (C-T and G-A transitions), additional PCRs and bi-directional sequencing were conducted, and a majority rule consensus applied to the four independent PCRs [53].

Consensus sequences were obtained from the alignment of fragments made in Geneious 5.6.3 [54].

### Data analysis

#### Genetic diversity

For microsatellite markers, we checked for scoring error due to stuttering, large allele drop-out, and null alleles using MICRO-CHECKER 2.2.3 [55]. Tests for Hardy-Weinberg proportions and genotypic disequilibrium for both historical and contemporary samples were conducted using exact probability tests with 1,000 iterations as implemented in GENEPOP 4.1 [56]. An unbiased estimate of the exact *P*-value was determined using a Markov chain method following the permutation algorithm of Guo and Thompson [57]. The significance threshold values for multiple statistical tests was adjusted using Bonferroni correction [58].

Indices of genetic diversity were calculated for both contemporary and museum samples. To assess the level of genetic variation in microsatellites, FSTAT was used to calculate means of allelic richness corrected for sample size [59], observed and expected heterozygosity (unbiased H, [60]) for each population. Statistical comparison between contemporary and historical samples for expected and observed heterozygosity, number of alleles and allelic richness were matched by loci and tested using a pairwise Wilcoxon signed-ranked test in R [61].

Mitochondrial diversity indices, such as number of haplotypes (*h*), nucleotide diversity (*π*; [62]), number of polymorphic sites (S) and haplotypic diversity (*Hd*) were computed in ARLEQUIN 3.5 [63] for contemporary, historical and fossil samples focusing on 450 bp of Domain I of the mtDNA CR.

We then constructed a temporal network to look at mitochondrial variation in kea through time using the R script TempNet v1.4. [64].

#### Historical population structure and assignment of historical samples

In order to compare historical and contemporary population structure, we examined the assignment of historical samples to contemporary populations. The rationale was that if the contemporary structure corresponded to the historical one, museum samples should cluster with samples of the same geographical origin. On the other hand, if the contemporary structure is the result of genetic drift caused by the recent population decline, historical samples should not cluster well with individuals of the same geographical origin or have more admixed membership. We first used STRUCTURE 2.2 [65, 66] to estimate the membership coefficient of each historical sample to one of three genetic clusters previously identified by Dussex et al. [45]. The dataset was analyzed using the LOCPRIOR option and the most probable value of *K* was estimated from 25 iterations of *K* values ranging from 2 to 4 (as the most likely *K* selected was 2 and 3 for contemporary data). The results obtained with STRUCTURE 2.2 were used as input in STRUCTURE HARVESTER [67] to produce the input files used for the following averaging step among runs. For the selected *K*-value the individuals‚ assignment coefficient (*q*) for each genetic cluster were averaged over the 10 runs with the lowest posterior probability using CLUMPP software [68]. Finally, the output obtained in CLUMPP was used and visualized in the program DISTRUCT [69].

#### Demographic history

For all analyses described below, we first considered the three genetic clusters (north, central and south) identified in Dussex et al. [45] (Fig. 1a) as advised by Broquet et al. [70] and Chikhi et al. [71] in order to avoid bias or the generation of a false bottleneck signal. Even though our historical data span nearly 60 years and may not represent a conventional population, we also considered the 14 historical samples (hereafter Historical Canterbury) independently for the analyses described below. Because kea are long-lived and have a long generation time, minimal bias caused by overlapping generations is expected.

Evidence for a genetic bottleneck was first evaluated using three moment-based methods: (i) the heterozygosity excess method implemented in the software BOTTLENECK v. 1.2.02 [72]. This approach assumes that a population that has gone through a recent reduction in effective size will show a high heterozygosity relative to heterozygosity at mutation-drift equilibrium [73]. We used the most appropriate TPM model for microsatellites [74] with 90% stepwise mutations as recommended by [75] as well as 95% of stepwise mutations and SMM as a comparison and chose a variance of 12 to encompass the range of multistep mutations observed in natural populations [74]; (ii) the mode-shift test implemented in the same software; (iii) the *M*-ratio test [75] using *M-P-Val* and *Critical_M* [75], which is more appropriate to detect genetic bottlenecks that occurred over relatively long periods of time (<100 generations). We used TPM mutation models with an amount of single step mutations of *p_s_* = 0.9 and *p_s_* = 0.78, 3.5 steps for multi-step mutations and pre-bottleneck *N_e_* values ranging from 500 and 5000. The mutation rate remained constant at μ of 5 × 10^-4^ [75]. The *M*-ratio was also calculated using only polymorphic loci to account for the upward bias in the *M*-ratio estimate, as implemented in ARLEQUIN 3.11 [63]. Secondly, in order to estimate variations of *N_e_* through time, we used the coalescent-based Bayesian method of Storz and Beaumont [76] implemented in the program MSVAR 1.3. This approach considers the demographic history of a closed population that increases or decreases exponentially from an historical size *N_H_* at *T* years in the past to the current *N_C_*, where the data are allele frequencies for unlinked microsatellites. These loci are assumed to have independent genealogies and to be evolving according to the strict single-step mutation model. Given the demographic parameters (contemporary population size, *N_C_*; historical population size *N_H_*; and the time interval *T*) and the mutation rate (μ), the genealogical history of the sampled data can be described using coalescent theory, and the posterior probability distribution of the genealogical and demographic parameters is estimated using Markov chain Monte Carlo (MCMC) simulations. We performed five simulations per dataset (north, central and south clusters and Historical Canterbury) with 2 × 10^9^ iterations with parameter values recorded every 1 × 10^5^ iterations resulting in 20,000 records. Prior distributions were set to the parameters of simulation 1 (S2 Table). After checking for chain convergence, the last 50% of the data from each chain was combined (50,000 sample points) and the mode and 90% highest posterior densities (HPD) were calculated for each parameter using the R-package Locfit 1.5–6 [77]. We then evaluated the strength of evidence for population expansion versus decline by calculating the Bayes factor for each of the models [78, 79] as described by Storz and Beaumont [80]. The Bayes factor indicates the following levels of support for the model; BF, 0.33 = false detection of contraction/expansion, 0.33–3 = no support, 3–10 = substantial support, and >10 = strong support [78]. We ran theses analyses for both exponential and linear models while putting more emphasis on the exponential model, which is more accurate at modeling recent population declines [81].

Finally, two different demographic scenarios were compared within an ABC framework [82] using DIYABC [83] for each dataset (north, central and south clusters and Historical Canterbury). This allowed us to determine whether bottlenecks occurred and also to estimate values for key parameters of interest such as pre- and post bottleneck effective population sizes and time and duration of the bottleneck. The first model described a population bottleneck occurring some 0 to 1,000 generations ago, a period largely encompassing the arrival of humans to New Zealand [84] if assuming a generation time of 7 to 10 years for kea (J. Kemp pers. comm.). The prior for historical *N_e_* (*N_H_*) was uniformly distributed between 10 and 2 × 10^5^, the upper bound encompassing 150,000 kea reported being killed between the 1870s and 1970s [44]. The prior for the contemporary *N_e_* (*N_C_*) was uniformly distributed between 10 and 10^4^. These values included the current and conservative population estimate of 1,000 to 5,000 birds [44] and also accounted for the difficulty in obtaining accurate estimates of contemporary population size. The duration of the bottleneck was uniformly distributed between 1 and 100 generations. For comparison, a second model of constant population size was also defined, in which *N_e_* was uniformly distributed through time for 0 to 1,000 generations and bounded between 10 and 2 × 10^5^ individuals. For the two models, a generalized stepwise mutation model [85] was implemented with a mean rate uniformly distributed between 1.00 × 10^-5^ and 1.00 × 10^-3^ substitutions/generations [86–88]. The posterior probabilities of each scenario were then estimated using both a direct and logistic regression approach providing both point estimated and 95% confidence intervals [89, 90]. The 10,000 datasets (1%) with the smallest Euclidian distances were then retained to build posterior parameter distributions.

Further details on tests of bottleneck detection using moment-based methods, variations of *N_e_* through time and demographic scenarios are available as S1 Supporting information.

## Results

### Genetic diversity

For museum samples, two microsatellite loci (Nmed31 and Strhab43) failed to amplify. After Bonferonni correction, no significant departure from Hardy-Weinberg equilibrium was observed and no tests for linkage disequilibrium were significant. Signs of null alleles were detected in single loci in the Kahurangi (Strhab43), Arthurs Pass (Nnot8) and Mt Cook (Nnot38) populations.

When comparing microsatellite genetic diversity among our historical and contemporary datasets, no extinct alleles were identified within the 15 skin samples (Table 2, S3 Table). Allelic richness was comparable in both datasets, being slightly lower in historical samples (Table 3). Mean number of alleles and expected heterozygosity were significantly different with historical samples showing lower diversity.

**Table 2:** Number of alleles sampled and allelic richness for contemporary and historical (1877–1934) samples.

	**Contemporary**				**Historical**			
**Locus**	**A**	**R_S_**	**H_O_**	**H_E_**	**A**	**R_S_**	**H_O_**	**H_E_**
Cfor0809‡	5	1.337	0.323	0.29	3	1.325	0.111	0.121
Nnot8*	3	1.488	0.52	0.46	2	1.495	0.444	0.329
Nnot14*	11	1.759	0.758	0.76	9	1.822	1	0.663
Nnot24*	4	1.492	0.408	0.40	3	1.384	0.778	0.429
Nnot37*	2	1.391	0.294	0.33	2	1.389	0.652	0.409
Nnot38*	2	1.241	0.283	0.27	1	1	0	0.000
Nnot39*	2	1.36	0.335	0.31	2	1.254	0.061	0.099
Nnot49*	3	1.504	0.483	0.46	2	1.508	0.833	0.495
Nnot49*	2	1.202	0.193	0.20	2	1.129	0.361	0.193
Strhab8°	2	1.351	0.253	0.24	2	1.349	0.182	0.132
Strhab13°	9	1.736	0.728	0.69	6	1.791	0.939	0.663
Strhab16°	2	1.336	0.258	0.27	2	1.271	0.367	0.252
Strhab25°	7	1.764	0.742	0.73	5	1.709	0.97	0.608
Strhab33°	7	1.776	0.695	0.72	7	1.836	0.939	0.630
Strhab35°	8	1.811	0.719	0.75	7	1.794	0.576	0.458

‡[91]

*[51]

°[92]

**Table 3:** Estimates of genetic diversity of kea microsatellite data within contemporary and historical (1877–1934) samples with the sample size for each region/population (n), observed heterozygosity (H_O_), expected heterozygosity (H_E_), allelic richness (R_S_), mean allele numbers (A) and *P*-values for H_E_ and R_S_.

**Sample**	**n**	**H_O_**	**H_E_** *	**R_S_**	**A** *
Historical (1877–1934)	15	0.548	0.365	1.470	3.667
Contemporary (2010–2011)	410	0.449	0.439	1.482	4.529

*significant difference with P <0.01

**Fig 2:**
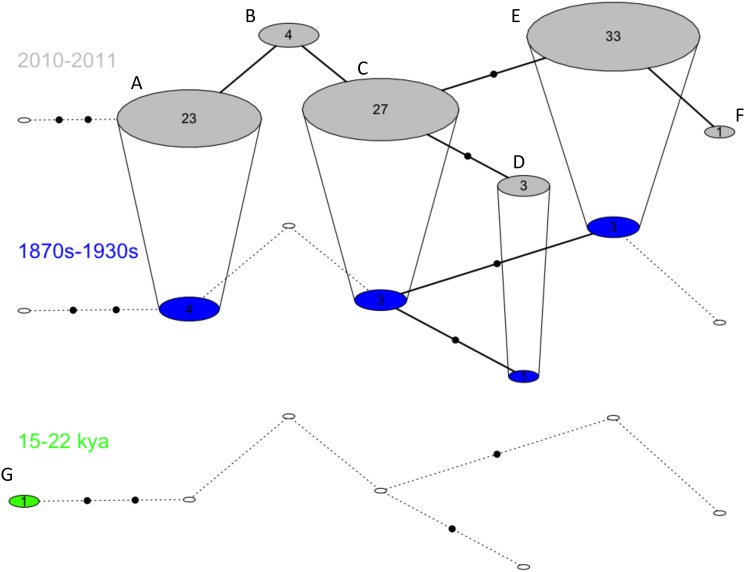
Temporal haplotype network displaying the relationships of kea haplotypes for 450 bp of the mitochondrial control region through time. Haplotypes are represented by circles and numbers represent sample size. Empty circles represent absent haplotypes for a given time period. Haplotypes found in multiple time period are connected by vertical lines. Within each time period, black dots represent one mutation.

Consensus sequences for the mtDNA CR for historical and fossil samples varied between 245 and 616 bp (Table 4). Three new substitution sites and two new haplotypes were found among fossil samples, while four of the haplotypes and five substitution sites found in contemporary samples were found among historical samples (Table 4). When considering only samples that amplified for 450 bp of the mtDNA CR (11 historical skins and 1 fossil bone), the temporal network was indicative of a loss of haplotypic diversity between the Late Glacial and contemporary period (late 1800s and 2000s) but further sampling of archeological, pre-human Holocene (0–11.6 kya), Late Glacial (11.6–14 kya) and Otiran Glacial (14–70 kya) material is required to confirm this trend. However, there is no recent indication of a loss of genetic diversity for mtDNA CR between the late 1800s to the present (Table 4, 5, Fig. 2).

**Table 4:** List of haplotypes for control region in kea with changes relative to haplotype A. Dots represent no change, dash unknown base due to partial sequence and boldened letters represent new haplotypes and mutations.

**Haplotype**	**n**	**Sample type**	**Total length of sequence obtained**	**Nucleotide site**									
				80	668	676	677	729	745	754	775	808	969
A	23	contemporary	1026	A	G	C	A	G	C	G	A	A	A
B	4	contemporary	1026	.	.	.	.	.	.	.	.	G	.
C	27	contemporary	1026	.	.	T	.	.	.	.	.	G	.
D	3	contemporary	1026	G	.	T	G	.	.	.	G	G	.
E	33	contemporary	1026	.	A	T	G	.	.	.	.	G	.
F	1	contemporary	1026	.	A	T	G	.	.	A	.	G	.
**G**	1	fossil^a^	450	-	.	.	G	**A**	**T**	.	.	.	-
**H**	3	fossil^b^	245	-	-	-	-	-	-	-	G	.	**G**
A	4	historical^c^	616	-	.	.	.	.	.	.	.	.	-
C	3	historical^d^	616	-	.	T	.	.	.	.	.	G	-
D	1	historical^e^	616	-	.	T	G	.	.	.	G	G	-
E	3	historical^f^	616	-	A	T	G	.	.	.	.	G	-

Sample reference numbers:

^a^NMNZ S.43574;

^b^NMNZ S.22664.1;

^c^CM AV84, CM AV89, CM AV91, AlM LB2466;

^d^AlM LB2469, NMW 12.209, NMW 12.211;

^e^CM AV89;

^f^CM AV2039, NHM 1927.12.18.125, NMW 49.760

**Table 5:** Estimates of genetic diversity (Domain 1 of the mitochondrial control region, 450 bp) within all, historical and fossil, and contemporary kea samples with the sample size for each dataset (n), number of observed haplotypes (*h*), number of polymorphic sites (S), haplotypic diversity (*Hd*), nucleotide diversity (*π*).

**Dataset**	**n**	***h***	**S**	***Hd***	**SD**	*π*	**SD**
Contemporary, Historical and Fossil*	103	7	8	0.728	0.018	0.00426	0.00271
Historical and Fossil*	12	5	7	0.813	0.070	0.00556	0.00362
Contemporary	91	6	6	0.721	0.020	0.00412	0.00264

*only samples for which 450 bp were amplified were considered here

### Historical population structure and assignment of historical samples

**Fig 3:**
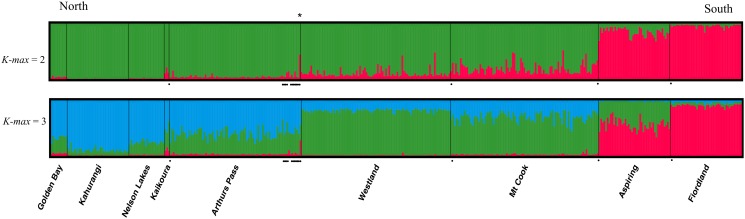
Individual clustering assignment for 410 contemporary and 15 historical Kea averaged for the 25 runs with the lowest ln(*K*) implemented in STRUCTURE for each of a different number of clusters (*K-max ∈* [2, 3]) in an admixture model. Historical samples are shown with a black dot and were grouped with samples of the same geographical location for the analysis. Sample NMW 12.211 referred in the text is shown with an asterisk.

When comparing historical and contemporary population structure using the Bayesian algorithm implemented in STRUCTURE, 14 individuals out of 15 clustered well with contemporary individuals from the same sampling locations. Of the 15 historical samples, 13 had high membership coefficients (*q* > 0.85) to their putative cluster of origin when considering two distinct genetic clusters (S4 Table). One sample originating from the Mt Cook population (1927.12.18.125) did not assign as strongly to its putative cluster of origin, with a *q*-value of 0.66 for the north cluster. However this is perhaps not surprising, as contemporary samples from the same location also show admixed ancestry (Fig. 3). Another historical sample originating from the central South Island (NMW 12.211, Lake Coleridge) showed shared membership to one of the two main clusters, with a *q*-value of 0.56 for the northern cluster and 0.44 for the south cluster (Fig. 3, S4 Table). When considering three genetic clusters, the Mt Cook sample (1927.12.18.125) showed admixed membership to the north and central cluster, but with the highest membership coefficient for its putative central cluster of origin (*q* = 0.69). Individual NMW 12.211 (Lake Coleridge, north cluster) showed again shared membership with *q*-values of 0.44, 0.28 and 0.28 for the north, central and south cluster respectively. Samples NMW 12.208 and NMW 49.760, putatively originating from the north cluster, also had admixed origins with *q*-values of 0.64 and 0.57 to the central cluster. However, those two samples had very high *q*-values of 0.94 and 0.88 to their cluster of origin when considering a scenario of two clusters. It is worth noting that because the distinction between the north and central cluster is not very strong and as gene flow is marked between these two clusters, shared membership between those two clusters is perhaps not surprising.

### Demographic history

Using the heterozygosity excess approach to bottleneck detection, TPM models with 90% and 95% of stepwise mutations only showed significant excess in heterozygotes for the central cluster (Table 6). The more statistically conservative SMM model [93] did not support a bottleneck in any of the clusters tested. Finally, there was no evidence in any population tested for a significant deviation from the normal L-shaped distribution of allele frequencies that could be expected in a population that is not in mutation-drift equilibrium.

**Table 6:** Bottleneck results implemented with three different tests for kea including *P*-values for a signed rank Wilcoxon test for heterozygous excess (one tail) with different models of mutations, the shifted mode test and the *M*-ratio method. For the heterozygous excess, TPM and SMM correspond to two-phase mutation model and single-step mutation model respectively. For the *M*-ratio method, *p_s_* stands for the proportion of sing-step mutations, *M_C_* for the critical *M* for a given theta where *θ* = 1 represents the initial (pre-decline) *N_e_* of 500 and *θ* = 10 an *N_e_* of 5000. *M*-ratio values that fall below the *M_C_* value are considered statistically significant at *α* = 0.05.

**Test**	**Parameter**		**North n = 142**	**Central n = 181**	**South n = 87**	**Historical Canterbury n = 15**
*H_e_* excess	TPM (90% SMM)		0.09	0.02	0.08	0.60
	TPM (95% SMM)		0.11	0.05	0.17	0.62
	SMM		0.18	0.14	0.36	0.74
Shifted Mode			No	No	No	No
*M*-Ratio	*p_s_* = 0.9	Δg = 3.5				
	*θ* = 1; *N_e_* = 500	*M_c_*	0.81	0.81	0.81	0.79
	*θ* = 10; *N_e_* = 5000	*M_c_*	0.75	0.76	0.74	0.65
		*M*-Ratio	0.76	0.79	0.86	0.83
	*p_s_* = 0.78	Δg = 3.5				
	*θ* = 1; *N_e_* = 500	*M_c_*	0.71	0.71	0.71	0.69
	*θ* = 10; *N_e_* = 5000	*M_c_*	0.70	0.71	0.68	0.56
		*M*-Ratio	0.76	0.79	0.86	0.83

When using the *M*-ratio approach, we found mixed results for signatures of a more ancient bottleneck (i.e. <100 gen.). Depending on the parameter set used, *M*-ratio values ranged from 0.76 to 0.86 (Table 6). These values are above or slightly below the critical value (*M_C_*) that represents the threshold under which a bottleneck is supported. We only found evidence of a bottleneck when using a *θ* value of 1, corresponding to a pre-decline *N_e_* of 500 and only for the north and central clusters.

Historical Canterbury and all three contemporary genetic clusters examined showed larger historical (*N_H_*) than contemporary (*N_C_*) effective population sizes for both demographic models (exponential and linear). Bayes factor values were >10 for all clusters and models indicating strong evidence for a population decline (Table 7). The ratio of the posterior distributions of contemporary and historical population sizes (r = *N_C_*/*N_H_*) clearly supported a population decline. Using an exponential model of population size change, the 90% highest posterior density (HPD) of the ratio r was of 0.0019–0.052, 0.053–0.068, 0.019–0.031 and of 0.0003–0.017 for Historical Canterbury, the north, central and south cluster samples respectively. These r values indicated that the contemporary *N_e_* is less than 10% of the historical *N_H_*.

**Table 7:** The Bayes Factor (BF) of each model, mode and 90% highest posterior density (in parentheses) for *N_C_*, *N_H_* and *T* for the demographic models implemented in MSVAR.

**Cluster**	**BF**	**model**	***N_H_***	***N_C_***	**Time (*T*)**
North	979	Exp	4,493 (1,112–19,085)	279 (76–1,016)	6,261 (1,208–34,300)
	5624	Linear	4,676 (1,198–17,124)	118 (24–521)	11,766 (2,126–67,321)
Central	inf	Exp	3,823 (1,111–14,791)	106 (22–470)	2,172 (354–12,843)
	inf	Linear	3,955 (1,122–14,701)	65 (4.4–461)	6,989 (1,122–41,399)
South	inf	Exp	4,620 (1,359–16,253)	28 (0.43–290)	271 (7–3,345)
	inf	Linear	4,677 (1,396–15,015)	11 (0.07–155)	2,417 (484–12,280)
Historical Canterbury	276	Exp	3,995 (889–20,427)	131 (1.8–1,067)	1,302 (20.2–39,058)
	624	Linear	4,266 (926–20,914)	103 (1–1,090)	6,708 (497–104,451)

**Fig 4:**
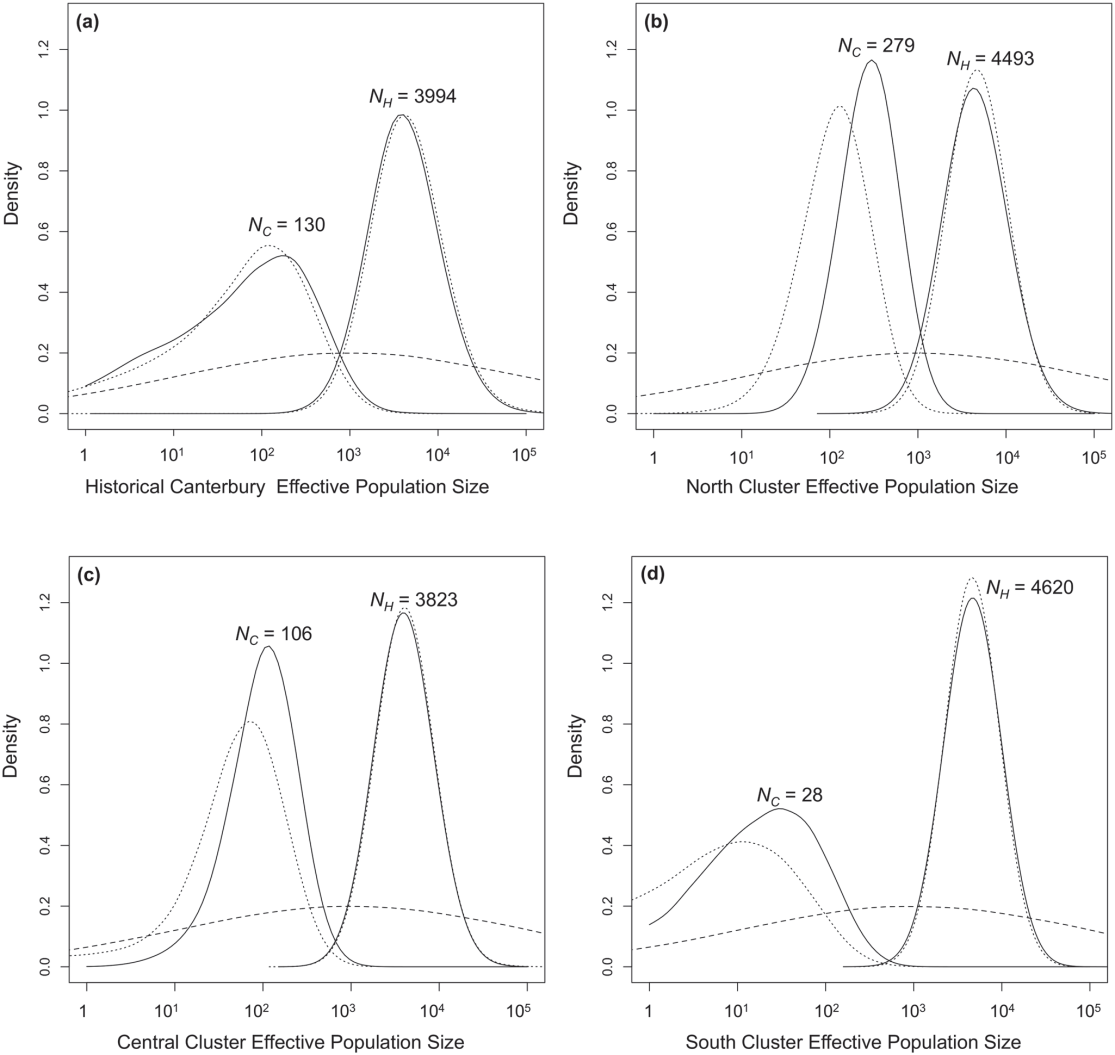
Prior and posterior distributions for the contemporary (*N_C_*) and historical (*N_H_*) effective population size (a) Historical Canterbury (b) north, (c) central (d) south clusters using both the exponential (full lines) and linear (dotted lines) models. The dashed line shows the prior distribution for *N_C_* and *N_H_*.

**Fig 5:**
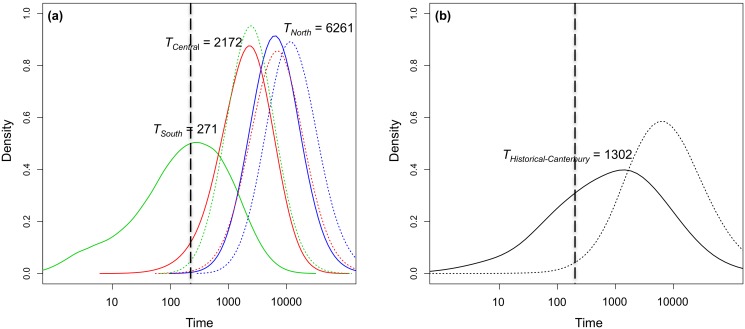
Posterior distribution of time of decline (***T***) (in years before present, assuming a generation time of seven years) (a) north, central and south clusters (b) Historical Canterbury using both the exponential (full lines) and linear (dotted lines) models. The vertical dotted line represents the approximate start of the governmental bounty scheme of the late 1800s.

The modes of the 90% HPD of the posterior distributions for historical effective population size (*N_H_*) for the exponential model were *N_historical-Canterbury_* = 3,995, *N_north_* = 4,493, *N_central_* = 3,823, and *N_south_* = 4,620 compared to modal values for contemporary effective population sizes (*N_C_*) of 131, 279, 106, and 28 respectively (Table 7, Fig. 4). Estimates of the time of population decline varied between populations. There was strong support for a population decline occurring well before human arrival in New Zealand in 1280 AD [84] for the Historical Canterbury, north and central clusters with *T_historical-Canterbury_* = 1,302 years before present [YBP], *T*_north_ = 6,261 and *T*_central_ = 2,172 YBP, while a more recent decline was supported for the south cluster with *T*_south_ = 271 YBP assuming a generation time of seven years (J. Kemp pers. comm.) (Table 7, Fig. 5).

Estimates for *N_H_* and *N_C_* for the linear model were similar to the exponential model (Table 7) while estimates for the timing of the decline were longer for the linear model for all genetic clusters (Table 7, Fig. 4). However, we focus here on the results of the exponential model because it is likely more realistic when modeling population dynamics [81].

Evaluation of two historical scenarios indicated that a model of population bottleneck best described the genetic data. This model received a posterior probability of 0.97, 0.99, 0.94 and 0.82 for the north, central, south clusters and Historical Canterbury respectively. Also, Type I and Type II error rates for the selection of the stable population size model ranged between 0.01 to 0.015 and between 0.02 to 0.03 respectively. Posterior estimation of parameters using values drawn from the 10,000 datasets closest to the observed ones for each cluster indicated that the effective population size has declined from 56,300, 10,800, 4,020 and 13,900 to 248, 380, 370 and 579 for the north, central, south clusters and Historical Canterbury respectively (Table 8). Timing of decline was estimated at 1,827, 6,461, 847 and 496.3 YBP assuming a generation time of seven years for the north, central, south clusters and Historical Canterbury respectively.

**Table 8:** The mode and 95% confidence interval for *N_C_*, *N_H_* and *T* (in number of generations) for the demographic models implemented in DIYABC for the scenario with the highest probability (population bottleneck).

**Cluster**	**Parameter**	**Prior range**	**Posterior mode**	**5%**	**95%**
North	*N_H_*	10 – 2 × 10^5^	56,300	12,200	423,000
	*N_C_*	10 – 10^4^	248	150	5,390
	*Time (T)*	0 – 1,000	261	71	924
	*Bottleneck duration*	1 – 100	86	6	96
	*μ rate (microsatellite)*	10^-6^ – 10^-3^	1 × 10^-5^	1 × 10^-5^	1.53 × 10^-5^
	*μ rate (mtDNA)*	10^-8^ – 10^-7^	9.93 × 10^-8^	3.49 × 10^-8^	9.94 × 10^-8^
Central	*N_H_*	10 – 2 × 10^5^	10,800	8840	378,000
	*N_C_*	10 – 10^4^	380	172	5,720
	*Time (T)*	0 – 1,000	923	114	963
	*Bottleneck duration*	1 – 100	78	6.1	95.7
	*μ rate (microsatellite)*	10^-6^ – 10^-3^	1 × 10^-5^	1 × 10^-5^	1.37 × 10^-5^
	*μ rate (mtDNA)*	10^-8^ – 10^-7^	9.70 × 10^-8^	2.71 × 10^-8^	9.9 × 10^-8^
South	*N_H_*	10 – 2 × 10^5^	4,020	2,530	32,400
	*N_C_*	10 – 10^4^	370	188	5,800
	*Time (T)*	0 – 1,000	121	60.8	895
	*Bottleneck duration*	1 – 100	6.83	4.2	94
	*μ rate (microsatellite)*	10^-6^ – 10^-3^	1 × 10^-5^	1 × 10^-5^	1.79 × 10^-5^
	*μ rate (mtDNA)*	10^-8^ – 10^-7^	9.79 × 10^-8^	2.51 × 10^-8^	9.9 × 10^-8^
Historical Canterbury	*N_H_*	10 – 2 × 10^5^	13,900	7,860	361,000
	*N_C_*	10 – 10^4^	579	214	6,820
	*Time (T)*	0 – 1,000	70.9	29.4	850
	*Bottleneck duration*	1 – 100	31.2	5.4	95.3
	*μ rate (microsatellite)*	10^-6^ – 10^-3^	1 × 10^-6^	1 × 10^-6^	1.17 × 10^-5^
	*μ rate (mtDNA)*	10^-8^ – 10^-7^	9.93 × 10^-8^	3.13 × 10^-8^	9.93 × 10^-8^

## Discussion

### Genetic diversity in historical and contemporary samples

No significant loss of genetic diversity was observed in sampled kea over the last 100 to 150 years, as no previously undetected haplotypes or alleles were identified among the historical samples. The higher diversity (mean number of alleles and expected heterozygosity) in contemporary samples was probably due to the paucity of samples available to our study with robust locality data. However, aDNA data shows two haplotypes and three new substitution sites found among fossil samples suggesting that a loss of genetic diversity might have occurred prior to the cull of the late 1800s. A larger sample size representing a wider time period between the Otiran Glacial to European arrival in New Zealand would be required to estimate the timing of such a decline and the extent of genetic diversity loss with more accuracy.

It is possible that our historical samples were collected after the peak of the government cull had already occurred. This is particularly true when considering the nature of the bottleneck. If for instance, the cull was rapid, of great intensity and resulted in populations being extirpated locally, it is quite likely that our historical sampling was not able to capture the effect of the bottleneck. If on the other hand, the cull lasted over a longer time span (> 10 years), we would be more likely to detect a loss of genetic diversity with our samples, as the commonly accepted date for the start of the government cull is 1860 [94]. However, we have little knowledge of the regional intensity and exact onset of this cull and it is possible that it varied among regions due to the regional difference in farming intensity.

Even if we cannot exclude the possibility of populations being extirpated locally in areas of high conflict with farmers, it is likely that some other populations could have persisted in more remote areas without farming and remained unaffected by the cull, thus maintaining historical levels of genetic diversity. Although this has never been reported, because of the dispersive [95, 96] and cognitive abilities of kea [34, 35], a temporary or permanent range shift might have occurred possibly in response to local persecution.

### Historical population structure and assignment of historical samples

Assignments of historical samples scored at 15 microsatellite loci did not suggest a different historical population structure, with most historical samples assigning well to their putative cluster of origin. This gives further support to the contemporary structure not resulting from genetic drift caused by the recent human-led population decline [45].

### Bottleneck detection using moment-based methods

The demographic histories of endangered and fluctuating populations is often complex. While conservationists often focus on the effect of human pressures on natural populations, the identification of a single decline event can be difficult, as the genetic variation of natural populations is influenced by multiple events varying in timing and nature. In this respect, kea is a good example as it has presumably been influenced by Pleistocene glaciations, climate change at the onset of the Holocene (11.6 kya), human colonization of New Zealand and by recent European persecution as well. Also, the usual rarity of kea fossils throughout New Zealand contrasting with a high abundance of remains in some sites [28] possibly reflects the effects of non-anthropogenic forces on the distribution of the species [30] while the lack of identified kea bones in middens [97] suggests that kea might not have experienced a range reduction resulting from over-hunting by Polynesians as in other endemic avian species (However the archeological record needs to be reassessed in light of the findings of this study). Kea history is therefore more complicated than previously thought since our results suggest that multiple forces may have influenced kea demography.

Moment-based methods of bottleneck detection yielded contrasting results. The *M*-ratio method did not detect any bottleneck while a signature of a bottleneck was detected in the central cluster using the heterozygote excess method and the most appropriate TPM model with 90% stepwise mutations [74]. As theory suggests, sign test under the IAM may be prone to (wrongly) detecting heterozygosity excess in non-bottlenecked populations [93]. Decreasing the proportion of stepwise mutations and thus going towards an IAM, therefore increased the significance of bottleneck detection in the samples we considered (Table 6). This became particularly obvious when using less than 90% stepwise mutation models (results not shown). While such a proportion is still likely, it can greatly vary among species, loci and alleles [75, 98]. Moreover, a well-known and recurrent problem with classical bottleneck detection methods is that one needs to make assumptions about microsatellite evolution [99]. Often, parameter values for the species of interest are unknown and must be borrowed from other closely-related species [75]. Thus, precise knowledge about the mutation rate of our markers is essential to be sure of the occurrence of a bottleneck. The lack of resolution of our markers in bottleneck detection might explain in part these contrasting results. Even though we are above the limit of number of loci recommended by the authors of BOTTLENECK (i.e. 8–10 polymorphic loci: [93]), an average of five alleles per loci might limit the power of the analyses.

The detection of a bottleneck signal in the central cluster is consistent with the fact that the McKenzie area was an easily accessible area and one of the first regions where intensive farming occurred [100–102]. There is no clear evidence for a variation in the intensity of the cull among regions, but if such variation in intensity of culling existed, it could explain why a bottleneck has only been detected in this region. The fact that results from both moment-based methods used here are not congruent, casts some doubt on this conclusion. One explanation for the observed pattern might be that the signal obtained with the heterozygote excess method is artificial. Bottleneck tests assume random mating (no population structure) and population closure (no gene flow). Non-random mating can produce genealogies that resemble bottlenecks, whereas gene flow is generally predicted to resemble recent expansion by introducing rare alleles [13, 73, 103]. It is therefore plausible that overlooking kea population substructure, although a rather weak one, could overshadow the effect of the bottleneck through an effect of immigration and replacement of genetic diversity lost locally [104].

### Detection of an ancient population decline

The modeling approaches implemented in MSVAR and DIYABC were the only methods that consistently identified population declines in the historical and contemporary samples examined. While mode estimates were slightly different, posterior distribution of parameters were overlapping among the two methods. However, the timing of this decline was not consistent with the reported cull of the late 1800s. Rather, our analysis identified a decline dating back a few hundreds of years ago (south cluster) to up to 6,000 years ago (north and central clusters) and suggests that kea could have experienced a population decline prior to human arrival in the northern part of its range at least. The finding of kea fossils from the Pleistocene in some areas of the South Island where kea are now absent such as central and eastern Otago [105], coastal North Otago [105, 106], Westland [30] and eastern Southland [107], further supports the possibility that kea went through at least one population contraction predating human arrival in New Zealand as suggested by [30]. The timing of this population decline is not unrealistic and although the present data does not allow the identification of the exact driver, a few events could have induced a population decline in kea.

Climate warming at the end of the last glacial period (Otiran Glacial) induced a change in environment in the South Island of New Zealand with the expansion of podocarp forest first [108, 109] and then its replacement by southern beech forest (∼5,000 YBP in the South) [108, 110, 111]. It is therefore possible that kea distribution could have changed with warming temperature and changing habitat. For instance, it has been shown that the extinct crested moa (*Pachyornis australis*) changed altitudinal, longitudinal and latitudinal ranges through the Late Quaternary in response to alterations in the distribution of suitable habitat [7]. The finding of a kea skeleton of Otiran age in the North Island [29] and apparent absence of North Island kea fossils from the Holocene first suggested that the species probably did not survive post-glacial warming and that the highly-forested North Island was not a suitable habitat for kea during the Holocene (however see below for new evidence). It is therefore possible that kea distribution shifted southwards towards the alpine areas of the South Island as kea were tracking their habitat. Also, the drier climate and more diverse vegetation in the eastern South Island [112, 113] may have provided a more favorable habitat for kea than the tall rain forests of Westland and the North Island. Such a difference in the timing of the decline in the South Island (i.e. ∼300 YBP in South and ∼6,000 YBP the North) can however not be fully be explained by the later southward establishment of forest in the South Island. Rather, fossils from the Holocene in lowland Canterbury [114] and newly discovered Holocene material from the North Island [32] suggest that the present distribution is relictual and reflects where kea survived human-caused extinctions and that they might have naturally occurred at lower altitude. This means that kea might not have been wiped out from the North Island because of climate warming during the Holocene but rather because of direct or indirect human activities such as hunting, predation by introduced mammals or habitat modification. This also suggests that the decline in effective population size in the South Island as shown by genetic data might not represent a sheer decline likely to drive the species to extinction but rather natural population fluctuations and range shifts in response to habitat change during the Holocene. During this period, kea populations could have remained stable with respect to the carrying capacity of their habitat, as has been shown for Australian mega- and meso-fauna [115, 116]. However, the rarity of Holocene specimens does not allow to detect a decline in response to Pleistocene-Holocene climate transition or to estimate its magnitude.

Another important aspect to consider is the possible impact of pre-European Maori on kea population dynamics. Estimates of the timing of decline predate Maori arrival in New Zealand (circa 1280 A.D., [84]) for the north and central clusters, but a much more recent decline for the south cluster (MSVAR, exponential model) seems concordant with Maori being linked with kea decline. While mentions of kea in Maori lore are rare [94, 117, 118], it is well known that Maori impacted on other New Zealand biotas through direct hunting [8, 119–121], habitat modification [122–124] and the introduction of rats [125, 126]. Since kea are scavengers [41, 127, 128], which has also been suggested by beak marks on fossil moa [114], it is possible that the extinction of moa and other birds potentially used as reliable food sources, could have somehow impacted kea population dynamics. The reason why such a recent decline is only observed on the south cluster and not in the other clusters examined remains unclear. The posterior distribution for the decline is rather wide, suggesting a possible methodological artifact and that a mode of ∼300 years should be taken with caution. However, if this estimation was not the result of a methodological artifact, it could be that kea had not been declining in the south prior human arrival and that they only recently declined.

### Non-detection of a recent population decline

Our results do not exclude the possibility of a recent (1800s to 1970s) human-induced population decline in kea and are consistent with the conclusion of Girod et al. [129] and the observation by Salmona et al. [130] that recent declines are not likely to be detected by MSVAR. However, a study on the heavily exploited Antarctic fur seal (*Arctocephalus gazella*) during the 1800’s suggests that ABC approaches are powerful enough to detect recent demographic declines [16]. The question therefore remains why a recent human-induced population decline was not detected with our ABC and moment-based methods. These incongruences among the various methods of bottleneck detection and the general pattern of non-detection of genetic bottleneck in the face of known demographic collapse is however not uncommon [19, 99, 103, 131–136] and a few factors can influence the efficiency of methodologies to detect genetic bottlenecks.

First of all, the detection of a bottleneck is highly dependent on the amount of genetic diversity present before the decline. If kea populations declined before human arrival as supported by the results of MSVAR and DIYABC, it is very likely that the inherent paucity in genetic variation following this ancient decline (300–6,000 years ago) could have precluded further genetic loss [73], making the detection of a recent decline impossible.

Secondly, the nature of the bottleneck is an important factor to consider. Even though classical methods of genetic bottleneck tests have reasonable power [73, 75, 93, 137], only very extreme population declines in *N_e_* (10- to 1000-fold) have been evaluated (e.g. Mexican Wolves, *Canis lupus mexicanus* [138]). If the extent of the population reduction of kea had been exaggerated and only moderate, it would not have been able to create a detectable signature in the form of heterozygote excess or *M*-ratio signal. With between 800 to 1,600 birds killed in a single season on some farming estates [139], it is possible that the bottleneck was short, rapid and intense, with most of the cull occurring in the first few years. Interestingly, large numbers of kea were still killed in the late 1940s [140], with nearly 7,000 birds killed over three years, suggesting kea might have in part recovered or still be maintained at high densities, probably thanks to new food sources such as wild ungulates or sheep [41, 128, 141]. Such a brief and extreme population decline and a possible quick population recovery may therefore not have left a signature of genetic bottleneck [15, 142, 143].

Thirdly, it is plausible that the cull was not homogeneous over the whole range of the species and that population density could have been significantly reduced mainly close to sheep stations, while populations in remote areas might have survived the cull [41]. Also, because of their cognitive behavior [34, 35] and high mobility [95, 96, 144], local populations could have easily shifted their range. It has been observed that the availability of new food sources might have attracted kea in numbers [41] and that groups of birds would move through the landscape, stopping to attack sheep and then disappear from the area for days or even weeks [95, 96]. Range shift could have also been induced by human persecution, as has been shown in other hunted species like wild boars [145]. It is thus quite likely that a combination of range-shift and immigration might have insured the maintenance of genetic diversity despite a localized population declines.

Finally, as has been suggested for long-lived species such as white-tailed eagle [146], Hawaiian petrel [19] or white-tailed black cockatoo [147], long generation time could lead to retention or buffering of genetic diversity because of a shorter effective time of exposure to bottleneck compared to a species with short generation time. Kea being able to reach up to 40 years of age in captivity [148] and with a generation time of 7 to 10 years (J. Kemp pers. comm.) could well fall in this category.

### Conservation implications

Although genetic signatures of historical contraction were detected, no recent bottleneck consistent with the report of an important cull was noted. This suggests that the cull was not strong enough to leave a genetic signature or that a combination of the factors discussed above reduced the probability of bottleneck detection. The discovery of additional mitochondrial diversity among fossil samples supports an ancient and possibly non-anthropogenic decline in kea, but further study of fossil samples is required to determine with more certainty the importance of habitat change during the Holocene on kea population dynamics. In a context of global climate change, our findings suggest that kea abundance could be further reduced by increasing temperatures either through associated habitat change, or through the effects of increased predator pressure in alpine habitats. While we cannot rule out the possibility that kea was once present in the lowlands and that it is now an alpine relict species because of habitat loss, persecution and predation, our study highlights the need to examine histories of endangered species and try and understand the respective anthropogenic and non-anthropogenic effects on population dynamics when proposing conservation guidelines.

The lack of detection of a recent genetic bottleneck associated with a documented human-induced demographic decline further highlights the need to interpret results of tests for population bottleneck with great caution. As failure to detect a genetic bottleneck does not exclude the possibility of a recent population contraction, our results further highlight the need to consider historical and fossil genetic diversity and effective population size estimates to develop efficient conservation recommendations for species of conservation concern.

While the species has been fully protected since 1986 [44], the introduction of mammalian predators in New Zealand has been an important driver of population decline in kea [25, 141]. It is also likely that the low genetic diversity in declining kea populations and ensuing inbreeding might contribute to the risk of extinction of the species [149]. We therefore recommend that further studies be done on the current levels of inbreeding and on future predicted loss of genetic diversity in extant kea populations.

## Supporting Information

S1 Supporting Information Details on tests for demographic history: bottleneck detection, fluctuations in *N_e_* and ABC scenarios. (PDF)

S1 Fig Diagram of amplicons used to sequence the partial mitochondrial control region in kea for toepads (top) and fossil samples (bottom). The left hand side of the figure represents the 5‚ end of the sequence.
(PDF)

S1 Table Primers used to amplify and sequence the partial mitochondrial control region in kea museum specimens (skins and fossil bones). (PDF)

S2 Table Prior and hyperprior parameters for runs of the Storz and Beaumont [14] analysis implemented in MSVAR. Columns 3–6 show the starting values for the mean and variance of the prior distributions. Columns 7–10 show the means and variances (and their means and variances) of the hyperprior distributions. Parameters listed are generation interval (g), contemporary *N_e_* (*N_C_*), historical *N_e_* (*N_H_*), mutation rate scaled in terms of contemporary population size (*θ*), and time (*T*). scale.
(PDF)

S3 Table Microsatellite allelic frequencies for contemporary and historical data. (PDF)

S4 Table Individual *q*-values for 15 historical Kea samples to each cluster identified in STRUCTURE when considering the two most likely clustering results for a two- (*K-max* = 2) or three-clusters (*K-max* = 3) scenario. Individuals with unusual assignments and referred to in the text are in bold.
(PDF)

S1 Data Historical microsatellite genotypes. (TXT)

S2 Data Historical and fossil mtDNA sequences. (TXT)
