# Supplementary material for: Ancient and Contemporary DNA Reveal a Pre-Human Decline but No Population Bottleneck Associated with Recent Human Persecution in the Kea (Nestor notabilis)
Source: PLoS One. 2015 Feb 26;10(2):e0118522. doi: 10.1371/journal.pone.0118522 (PMC4342260; doi:10.1371/journal.pone.0118522)
Supplement: S1 Supporting Information — (PDF) [file pone.0118522.s001.pdf]

S1 Supporting Information

Demographic history

We first used the software BOTTLENECK v. 1.2.02 [1] to document reductions in population size that are relatively recent. Because the heterozygosity excess is transient, it will only persist for $0.2 - 4N_e$ generations after the bottleneck [1, 2]. The TPM has been shown to be the most appropriate model for microsatellites [3]. Even though a TPM with 90% stepwise mutations was recommended as a conservative model of microsatellite evolution [4], we also ran the model with 95% of stepwise mutations and SMM as a comparison. We also used a variance of 12 to encompass the range of multistep mutations observed in natural populations [3] and 10,000 iterations for each mutation model. To determine if the number of loci exhibiting heterozygosity excess was significant, the one-tailed Wilcoxon signed rank test for heterozygote excess was applied.

Second, using the same software, we tested the distribution of allele frequencies to determine whether a bottleneck-induced mode shift has occurred. A mode shift is a transient distortion in the distribution of allele frequencies such that the frequency of alleles at low frequencies become lower than the frequency of alleles in an intermediate allele frequency class [5].

Third, in order to detect genetic bottlenecks that occurred over relatively long periods of time (<100 generations), we used the M-ratio test [4]. This test is based on the ratio of the observed number of microsatellite alleles and the range of allele sizes, such that the M-ratio decreases in bottlenecked populations when alleles are randomly lost as a result of genetic drift [4]. We used the software *M-P-Val* to calculate M and software *Critical_M* to determine the cut-off value

for statistical significance [4]. *Critical_ M* simulates an equilibrium distribution of M (M_{C}) in a constant population assuming values for three parameters: θ, the parameter based on effective population size prior the bottleneck and mutation rate; Δg, the average size of non one-step mutations; and p_s, the proportion of one-step mutations. A reduction in effective size is suggested when the M-ratio falls below the simulated M_{C}. We simulated two sets of parameters values: the first is a reasonable parameterization of the two-phase mutation model as noted by Garza and Williamson [4] with $p_s = 0.9$ and $\Delta g = 3.5$; the second set of parameter values, with a lower proportion of stepwise mutations $p_s = 0.78$ and $\Delta g = 3.5$ is less realistic [4], but still useful for comparison. It is thus expected that the loci used here would follow a mutation model in between those two extremes. We also tested two values of θ parameter (1 and 10) corresponding to a pre-decline N_e of 500 and 5,000, respectively, and the mutation rate μ was held constant at 5×10^{-4} [4]. Although smaller values of θ will increase the value of M_{C}, we set the value of θ at a maximum of 10, because θ is population specific. In order to account for the upward bias in the M-ratio estimate, the M-ratio was also calculated using only polymorphic loci, as implemented in Arlequin 3.11 [6].

It is worth noting that the heterozygote excess and mode-shift tests detect more recent bottlenecks, while the M-ratio method is suited to detecting older bottlenecks (<100 generations) [4]. Heterozygote excess is reduced rapidly as a population expands and reaches a new migration–drift equilibrium ($< 4\ N_e$ generations), while gaps in the allele frequency spectrum can only be filled in by new mutations or migration [7].

In order to assess changes in N_e, we used the coalescent-based Bayesian method of Storz and Beaumont [8] implemented in the program MSVAR 1.3. We initially

conducted six independent simulations of model varying the prior and hyperprior distributions with a range of biologically realistic distribution values to examine their effect on the posterior distributions. These variations of the priors had little effect on the posterior distribution of the models so prior distributions for all other analyses were set to the parameters of simulation one (Table S2). In order to check for the convergence of model we ran the simulations five times for each dataset considered. Each simulation was performed for 2×10^9 iterations with parameter values recorder every 1×10^5 iterations resulting in 20,000 records. We discarded the first 10% of recorded values for each chain (when simulations may not have stabilized), and processed the data using the computer program BOA version 1.1.4 [9] for R version 2.3.1 [10]. The Brooks, Gelman and Rubin Convergence diagnostic tests were done using boa on all the data chains to check statistically for convergence (Gelman & Rubin 1992; Brooks & Gelman 1998). Convergence of the chains is demonstrated where the corrected scale reduction factor output approximates a value of 1, indicating the samples have arisen from a stationary distribution [9]. The potential scale reduction factors for all three parameters were approximately 1 ($\mathrm{Log}_{10} N_C = 1.002$; $\mathrm{Log}_{10} N_H = 1.07$; $\mathrm{Log}_{10} T = 1.06$) for each sample analyzed, providing statistical evidence for convergence of the chains. Thereafter, the last 50% of the data from the five chains were combined (50 000 sample points) and the mode and 90% highest posterior densities (HPD) were calculated for each parameter using the R-package Locfit 1.5–6 [11]. We evaluated the strength of evidence for population expansion versus decline by calculating the Bayes factor for each of the models [12, 13] as described by Storz and Beaumont [14] and Girod *et al.* [15]. The Bayes factor indicates the following levels of support for the model; BF, 0.33 = false detection of contraction/ expansion, 0.33–3 = no

support, 3–10 = substantial support, and >10 = strong support [13]. We ran theses analyses for both exponential and linear models. However, an exponential model of population size change is expected to be more accurate at modeling recent population declines [16].

Finally, for the comparison of population scenarios using an ABC approach [17], we simulated 1,00,000 genetic datasets for each scenario (bottleneck and constant population size), with the demographic and marker parameters described above. These simulations were independently carried out considering the three clusters previously identified by clustering analyses [18]. Seven summary statistics were generated for the observed and simulated datasets: mean gene diversity [19], the mean number of alleles across loci, mean allele size variance across loci and Garza and Williamson's M [4] for microsatellite data and the number of haplotypes, the number of segregating sites and Tajima's D [20] for mtDNA. For each population or cluster tested, normalized Euclidian distance was calculated between the observed dataset and each of the simulated datasets using the local linear regression method of Beaumont et al. [17]. The 10,000 simulated datasets with the smallest Euclidian distances were then retained to build posterior parameter distribution, which were smooth weighted using the Locfit function within R version 2.9.2 [10]. The posterior probabilities of each scenario were first estimated by taking the 500 simulated data sets closest to the observed data set and calculating the proportion that belong to each scenario (direct approach) and secondly using a logistic regression approach on the closest 1% of data sets to the observed data, providing both point estimated and 95% confidence intervals [21, 22]. Statistical measures of performance and Type I and Type II error rates were also calculated as a method of model checking [6]. The above analyses were implemented within the DIYABC

V 1.0.4 software package [22, 23].

Additionally, we performed a model checking step implemented in DIYABC V 1.0.4 and tested the "goodness-of-fit" of the chosen scenario to the observed data, as advised by Cornuet et al.[23]. This model checking procedure consists of simulating the pseudo-observed data sets with parameters drawn from the posterior parameter distribution of the considered scenario and positioning the summary statistics of the observed data in the summary statistic distribution of the pseudo-observed data. The scenario is then considered suitable if the observed data summary statistics are included in the confidence interval drawn from pseudo-observed data [23].

References

[1] Piry S, Luikart G, Cornuet JM (1999) BOTTLENECK: A computer program for detecting recent reductions in the effective population size using allele frequency data. Journal of Heredity 90: 502–503.

[2] Luikart G, Cornuet JM (1998) Empirical evaluation of a test for identifying recently bottlenecked populations from allele frequency data. Conservation Biology 12: 228–237.

[3] Dirienzo A, Peterson AC, Garza JC, Valdes AM, Slatkin M, et al. (1994) Mutational Processes of Simple-Sequence Repeat Loci in Human-Populations. Proceedings of the National Academy of Sciences of the United States of America 91: 3166–3170.

[4] Garza JC, Williamson EG (2001) Detection of reduction in population size using data from microsatellite loci. Molecular Ecology 10: 305–318.

[5] Luikart G, Allendorf FW, Cornuet JM, Sherwin WB (1998) Distortion of allele frequency distributions provides a test for recent population bottlenecks. Journal of Heredity 89: 238–247.

[6] Excoffier L, Estoup A, Cornuet JM (2005) Bayesian analysis of an admixture model with mutations and arbitrarily linked markers. Genetics 169: 1727–1738.

[7] Williamson-Natesan EG (2005) Comparison of methods for detecting bottlenecks from microsatellite loci. Conservation Genetics 6: 551–562.

[8] Storz JF, Beaumont MA (2002) Testing for genetic evidence of population expansion and contraction: An empirical analysis of microsatellite DNA variation using a hierarchical Bayesian model. Evolution 56: 154–166.

[9] Smith BJ (2007) boa: An R package for MCMC output convergence assessment and posterior inference. Journal of Statistical Software 21: 1–37.

[10] R Development Team (2005) No Title. R: A Language and Environment for Statistical Computing : R Foundation for Statistical Computing, Vienna, Au.

[11] Loader C (2007) Locfit: Local regression, likelihood and density estimation. R Package Version: 1.5 - 4 .

[12] Kass RE, Raftery AE (1995) Bayes Factors. Journal of the American Statistical Association 90: 773–795

[13] Jeffreys H (1961) Theory of probability. Oxford, UK: Oxford University Press 447p.

[14] Storz JF, Beaumont MA, Alberts SC (2002) Genetic evidence for long-term population decline in a savannah-dwelling primate: Inferences from a hierarchical Bayesian model. Molecular Biology and Evolution 19: 1981–1990.

[15] Girod C, Vitalis R, Leblois R, Freville H (2011) Inferring Population Decline and Expansion From Microsatellite Data: A Simulation-Based Evaluation of the Msvar Method. Genetics 188: 165–179.

[16] Beaumont MA (1999) Detecting population expansion and decline using microsatellites. Genetics 153: 2013–2029.

[17] Beaumont MA, Zhang WY, Balding DJ (2002) Approximate Bayesian computation in population genetics. Genetics 162: 2025–2035.

[18] Dussex N, Wegmann D, Robertson BC (2014) Postglacial expansion and not human influence best explains the population structure in the endangered kea (Nestor notabilis). Molecular Ecology 23: 2193–2209.

[19] Nei M (1987) Molecular evolutionary genetics.

[20] Tajima F (1989) Statistical method for testing the neutral mutation hypothesis by DNA polymorphism. Genetics 123: 585–595.

[21] Fagundes NJR, Ray N, Beaumont M, Neuenschwander S, Salzano FM, et al. (2007) Statistical evaluation of alternative models of human evolution. Proceedings of the National Academy of Sciences of the United States of America 104: 17614–17619.

[22] Cornuet JM, Santos F, Beaumont MA, Robert CP, Marin JM, et al. (2008) Inferring population history with DIY ABC: a user-friendly approach to approximate Bayesian computation. Bioinformatics 24: 2713–2719.

[23] Cornuet JM, Ravigne V, Estoup A (2010) Inference on population history and model checking using DNA sequence and microsatellite data with the software DIYABC (v1.0). BMC Bioinformatics 11.
