# Supplementary figures and images for: Ancient and Contemporary DNA Reveal a Pre-Human Decline but No Population Bottleneck Associated with Recent Human Persecution in the Kea (Nestor notabilis)
Source: PLoS One. 2015 Feb 26;10(2):e0118522. doi: 10.1371/journal.pone.0118522 (PMC4342260; doi:10.1371/journal.pone.0118522)

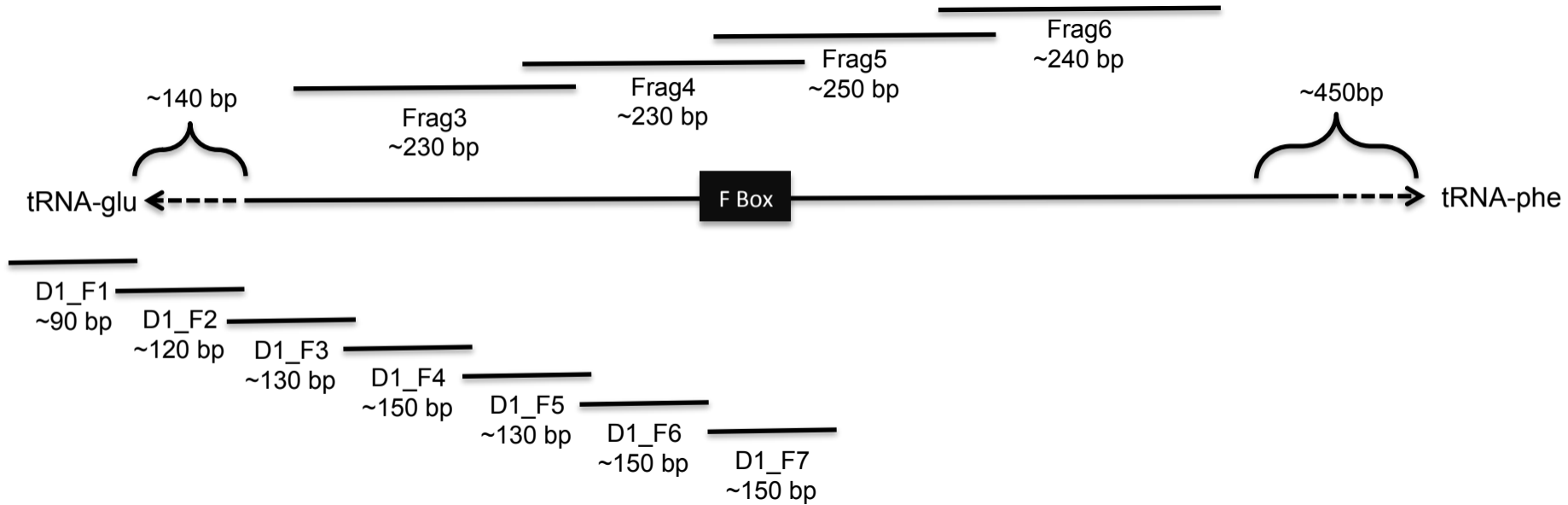

Supplement: S1 Fig — The left hand side of the figure represents the 5‚ end of the sequence. (PDF) [file pone.0118522.s002.pdf]
