# Supplementary material for: Ancient and Contemporary DNA Reveal a Pre-Human Decline but No Population Bottleneck Associated with Recent Human Persecution in the Kea (Nestor notabilis)
Source: PLoS One. 2015 Feb 26;10(2):e0118522. doi: 10.1371/journal.pone.0118522 (PMC4342260; doi:10.1371/journal.pone.0118522)
Supplement: S1 Table — (PDF) [file pone.0118522.s003.pdf]

Fragment	Primer	Sequence (5'-3')
Toepad samples		
3	Frag 3_F	TCGAATTCAAAGTGCTTTTGC
	Frag 3_R	TTCCGTATGGCAAATATATGAATG
4	Frag 4_F	TCACGGAATGGAGACTGTCA
	Frag 4_R	CTTACACCGGGTTGCTGATT
5	Frag 5_F	AAGCTGCATTCGTCCTACCA
	Frag 5_R	CAAATAGCGAACCAGATGACTC
6	Frag 6_F	CTCTTTTGCGCCTCTGGTT
	Frag 6_R	TACAGGTGCGCCTGGAAT
Fossil bone samples		
1	D1_F1_F	TGGCCTGAAAAACCATCGTTG
	D1_F1_R	TTGAGGTGAGGATTTGGGGG
2	D1_F2_F	TGTCAACTCAACTATAAGAACAGCC
	D1_F2_R	AGCAAAAGCACTTTGAATTCGA
3	D1_F3_F	TGGACTTAATTTTGAATTCAAATTCGA
	D1_F3_R	TCTTGATGCATAAATTATTTAGTCCCT
4	D1_F4_F	TCAAAGTGCTTTTGCTTTTATGTAAT
	D1_F4_R	TGAAGCGAAACAGGGGAAAAG
5	D1_F5_F	TTTATGCATCAAGACATGATTTCCA
	D1_F5_R	TGGTGTCCATTGTTTGTTTGGG
6	D1_F6_F	TGGAGACTGTCATGACAAAAGACA
	D1_F6_R	GTAGGACGAATGCAGCTTGC
7	D1_F7_F	TACCTCCCACACATGGCAAC
	D1_F7_R	ACGCTAGTAGTGTAGGACCTT
