# Supplementary material for: Ancient and Contemporary DNA Reveal a Pre-Human Decline but No Population Bottleneck Associated with Recent Human Persecution in the Kea (Nestor notabilis)
Source: PLoS One. 2015 Feb 26;10(2):e0118522. doi: 10.1371/journal.pone.0118522 (PMC4342260; doi:10.1371/journal.pone.0118522)
Supplement: S2 Table — Columns 3–6 show the starting values for the mean and variance of the prior distributions. Columns 7–10 show the means and variances (and their means and variances) of the hyperprior distributions. Parameters listed are generation interval (g), contemporary Ne (NC), historical Ne (NH), mutation rate scaled in terms of contemporary population size (θ), and time (T). scale. (PDF) [file pone.0118522.s004.pdf]

Run #	g	log(N_C)	log(N_H)	log(θ)	log(T)	log(N_C)	log(N_H)	log(θ)	log(T)
1	7	3 2	3 2	-3.5 1	3 1	3 2 0 0.5	3 2 0 0.5	-3.5 0.25 0 0.5	3 2 0 0.5
2	7	4 1	5 1	-3.5 1	5 1	3 2 0 0.5	3 2 0 0.5	-3.5 0.25 0 0.5	3 2 0 0.5
3	7	4 1	6 1	-3.5 1	5 1	3 2 0 0.5	3 2 0 0.5	-3.5 0.25 0 0.5	3 2 0 0.5
4	7	4 1	5 1	-3.5 1	3 1	3 2 0 0.5	3 2 0 0.5	-3.5 0.25 0 0.5	3 2 0 0.5
5	7	4 1	5 1	-3.5 1	2 1	3 2 0 0.5	3 2 0 0.5	-3.5 0.25 0 0.5	3 2 0 0.5
6	7	3 1	5 1	-3.5 1	2 1	3 2 0 0.5	3 2 0 0.5	-3.5 0.25 0 0.5	3 2 0 0.5
