# Supplementary material for: Ancient and Contemporary DNA Reveal a Pre-Human Decline but No Population Bottleneck Associated with Recent Human Persecution in the Kea (Nestor notabilis)
Source: PLoS One. 2015 Feb 26;10(2):e0118522. doi: 10.1371/journal.pone.0118522 (PMC4342260; doi:10.1371/journal.pone.0118522)
Supplement: S3 Table — (PDF) [file pone.0118522.s005.pdf]

Locus	Allele/n	Golden Bay	Kahurangi	Nelson Lakes	Kaikoura	Arthurs Pass	Westland	Mt Cook	Aspiring	Fiordland	Museum samples
**Nnot14**	**N**	10	38	22	3	69	92	89	44	43	13
	**198**	0.000	0.000	0.000	0.000	0.007	0.000	0.000	0.034	0.081	0.000
	**202**	0.000	0.000	0.000	0.000	0.000	0.005	0.017	0.023	0.012	0.000
	**208**	0.200	0.118	0.091	0.000	0.094	0.168	0.169	0.068	0.093	0.077
	**214**	0.000	0.039	0.091	0.000	0.029	0.022	0.051	0.011	0.081	0.038
	**218**	0.250	0.342	0.432	0.667	0.529	0.533	0.438	0.398	0.279	0.385
	**224**	0.350	0.039	0.045	0.167	0.058	0.049	0.084	0.182	0.140	0.154
	**228**	0.050	0.171	0.114	0.000	0.080	0.076	0.112	0.045	0.081	0.038
	**234**	0.050	0.237	0.205	0.000	0.087	0.049	0.073	0.080	0.093	0.115
	**238**	0.050	0.039	0.000	0.000	0.072	0.016	0.056	0.011	0.047	0.038
	**244**	0.050	0.013	0.023	0.000	0.043	0.082	0.000	0.034	0.081	0.115
	**248**	0.000	0.000	0.000	0.167	0.000	0.000	0.000	0.114	0.012	0.038
**Strhab16**	**N**	10	38	22	3	69	92	89	44	43	13
	**142**	0.100	0.053	0.182	0.000	0.196	0.217	0.225	0.273	0.349	0.154
	**146**	0.900	0.947	0.818	1.000	0.804	0.783	0.775	0.727	0.651	0.846
**Strhab8**	**N**	10	37	21	1	57	87	81	38	38	14
	**112**	1.000	0.905	0.833	1.000	0.886	0.638	0.698	0.724	0.895	0.786
	**120**	0.000	0.095	0.167	0.000	0.114	0.362	0.302	0.276	0.105	0.214
**Strhab33**	**N**	10	38	22	3	69	92	89	44	43	14
	**164**	0.000	0.000	0.000	0.000	0.014	0.022	0.011	0.284	0.116	0.107
	**166**	0.250	0.263	0.205	0.500	0.297	0.451	0.343	0.216	0.244	0.214
	**168**	0.200	0.066	0.114	0.000	0.130	0.223	0.270	0.273	0.209	0.179
	**170**	0.400	0.355	0.295	0.333	0.319	0.092	0.191	0.136	0.384	0.286
	**172**	0.000	0.000	0.000	0.000	0.000	0.000	0.000	0.011	0.035	0.036
	**174**	0.150	0.303	0.364	0.167	0.152	0.190	0.146	0.068	0.000	0.143
	**176**	0.000	0.013	0.023	0.000	0.087	0.022	0.039	0.011	0.012	0.036
**Strhab13**	**N**	10	38	22	3	69	92	89	44	43	14
	**117**	0.200	0.211	0.114	0.167	0.196	0.245	0.208	0.284	0.221	0.286
	**121**	0.000	0.000	0.000	0.000	0.000	0.000	0.000	0.000	0.023	0.000
	**123**	0.050	0.039	0.182	0.000	0.138	0.092	0.185	0.136	0.047	0.071
	**125**	0.000	0.000	0.000	0.000	0.000	0.000	0.000	0.023	0.093	0.000
	**128**	0.200	0.026	0.068	0.167	0.029	0.043	0.084	0.057	0.058	0.071
	**130**	0.050	0.303	0.273	0.000	0.130	0.071	0.197	0.159	0.372	0.286
	**132**	0.500	0.421	0.364	0.667	0.507	0.549	0.326	0.341	0.174	0.250
	**134**	0.000	0.000	0.000	0.000	0.000	0.000	0.000	0.000	0.012	0.036
**Strhab25**	**N**	10	38	21	3	67	92	89	44	43	14
	**177**	0.200	0.250	0.214	0.000	0.239	0.397	0.388	0.307	0.477	0.393
	**181**	0.000	0.000	0.000	0.000	0.000	0.005	0.000	0.068	0.093	0.000
	**185**	0.500	0.263	0.286	0.500	0.194	0.304	0.309	0.261	0.221	0.357
	**189**	0.050	0.066	0.238	0.000	0.201	0.207	0.180	0.261	0.151	0.179
	**193**	0.100	0.132	0.119	0.167	0.007	0.011	0.000	0.000	0.000	0.000
	**197**	0.150	0.118	0.095	0.333	0.216	0.022	0.034	0.034	0.035	0.036
	**201**	0.000	0.171	0.048	0.000	0.142	0.054	0.090	0.068	0.023	0.036

Locus	Allele/n	Golden Bay	Kahurangi	Nelson Lakes	Kaikoura	Arthurs Pass	Westland	Mt Cook	Aspiring	Fiordland	Museum samples
**Nnot24**	**N**	8	35	22	3	67	85	86	43	43	15
	**159**	0.000	0.000	0.000	0.000	0.000	0.000	0.000	0.035	0.151	0.033
	**163**	0.438	0.100	0.114	0.000	0.090	0.288	0.209	0.372	0.279	0.200
	**167**	0.563	0.843	0.841	1.000	0.888	0.618	0.715	0.477	0.326	0.767
	**171**	0.000	0.057	0.045	0.000	0.022	0.094	0.076	0.116	0.244	0.000
**Strhab35**	**N**	10	37	22	3	67	91	88	44	42	14
	**149**	0.050	0.162	0.205	0.167	0.172	0.159	0.148	0.227	0.274	0.143
	**155**	0.100	0.095	0.068	0.000	0.216	0.203	0.205	0.409	0.167	0.250
	**158**	0.100	0.014	0.136	0.333	0.119	0.143	0.193	0.250	0.333	0.036
	**160**	0.450	0.378	0.318	0.500	0.269	0.220	0.239	0.091	0.214	0.357
	**163**	0.150	0.081	0.045	0.000	0.037	0.011	0.023	0.011	0.000	0.036
	**165**	0.000	0.027	0.000	0.000	0.000	0.000	0.011	0.000	0.000	0.000
	**167**	0.100	0.230	0.227	0.000	0.172	0.264	0.182	0.011	0.012	0.143
	**169**	0.050	0.014	0.000	0.000	0.015	0.000	0.000	0.000	0.000	0.036
**Cfor0809**	**N**	10	38	22	3	68	90	85	36	43	11
	**182**	0.000	0.000	0.000	0.000	0.007	0.000	0.000	0.000	0.035	0.000
	**188**	0.950	0.947	0.955	0.667	0.897	0.733	0.735	0.681	0.733	0.818
	**192**	0.050	0.053	0.045	0.333	0.081	0.261	0.218	0.306	0.163	0.136
	**196**	0.000	0.000	0.000	0.000	0.015	0.006	0.047	0.014	0.023	0.045
	**200**	0.000	0.000	0.000	0.000	0.000	0.000	0.000	0.000	0.047	0.000
**Nnot37**	**N**	10	37	21	3	69	92	87	43	42	14
	**247**	0.250	0.392	0.310	0.000	0.333	0.293	0.259	0.140	0.131	0.250
	**249**	0.750	0.608	0.690	1.000	0.667	0.707	0.741	0.860	0.869	0.750
**Nnot38**	**N**	10	38	22	3	69	92	89	44	43	13
	**213**	0.800	0.763	0.773	0.833	0.884	0.864	0.910	0.920	0.791	1.000
	**216**	0.200	0.237	0.227	0.167	0.116	0.136	0.090	0.080	0.209	0.000
**Nnot39**	**N**	10	38	22	3	68	92	88	44	43	14
	**223**	0.750	0.592	0.659	1.000	0.824	0.723	0.756	0.920	0.814	0.857
	**225**	0.250	0.408	0.341	0.000	0.176	0.277	0.244	0.080	0.186	0.143
**Nnot43**	**N**	10	38	22	3	69	91	86	44	43	15
	**173**	0.400	0.316	0.341	0.833	0.486	0.582	0.512	0.443	0.384	0.567
	**177**	0.600	0.684	0.659	0.167	0.514	0.418	0.471	0.545	0.616	0.433
	**181**	0.000	0.000	0.000	0.000	0.000	0.000	0.017	0.011	0.000	0.000
**Nnot49**	**N**	10	37	22	3	68	91	89	43	42	15
	**206**	0.950	0.851	0.886	0.833	0.875	0.901	0.927	0.977	0.714	0.933
	**208**	0.050	0.149	0.114	0.167	0.125	0.099	0.073	0.023	0.286	0.067
**Nnot8**	**N**	9	38	22	3	69	92	89	44	43	14
	**164**	0.389	0.539	0.477	0.667	0.457	0.560	0.612	0.852	0.651	0.607
	**166**	0.000	0.000	0.000	0.000	0.000	0.005	0.000	0.000	0.012	0.000
	**168**	0.611	0.461	0.523	0.333	0.543	0.435	0.388	0.148	0.337	0.393
