# Supplementary material for: Ancient and Contemporary DNA Reveal a Pre-Human Decline but No Population Bottleneck Associated with Recent Human Persecution in the Kea (Nestor notabilis)
Source: PLoS One. 2015 Feb 26;10(2):e0118522. doi: 10.1371/journal.pone.0118522 (PMC4342260; doi:10.1371/journal.pone.0118522)
Supplement: S4 Table — Individuals with unusual assignments and referred to in the text are in bold. (PDF) [file pone.0118522.s006.pdf]

***K-max* = 2**

Sample	Sampling location	Putative cluster of origin	Q values	
			North	South
LB2467	Sumner	North	0.98	0.02
NMW 12.208	Arthurs	North	0.94	0.06
NMW 12.209	Arthurs	North	0.96	0.04
LB2466	Arthurs	North	0.97	0.03
NMW 49.760	Porters	North	0.88	0.12
AV84	Coleridge	North	0.96	0.04
AV86	Coleridge	North	0.92	0.08
AV88	Coleridge	North	0.97	0.03
AV89	Coleridge	North	0.86	0.14
AV90	Coleridge	North	0.96	0.04
AV91	Coleridge	North	0.98	0.02
**NMW 12.211**	**Coleridge**	**North**	**0.56**	**0.44**
AV2039	Tekapo	North	0.86	0.14
**1927.12.18.125**	**Cook**	**North**	**0.66**	**0.34**
LB2469	Milford	South	0.02	0.98

***K-max* = 3**

Sample	Sampling location	Putative cluster of origin	Q values		
			North	Central	South
LB2467	Sumner	North	0.77	0.22	0.02
**NMW 12.208**	**Arthurs**	**North**	**0.34**	**0.64**	**0.02**
NMW 12.209	Arthurs	North	0.63	0.35	0.03
LB2466	Arthurs	North	0.56	0.41	0.03
**NMW 49.760**	**Porters**	**North**	**0.39**	**0.57**	**0.04**
AV84	Coleridge	North	0.59	0.38	0.03
AV86	Coleridge	North	0.61	0.34	0.05
AV88	Coleridge	North	0.61	0.37	0.02
AV89	Coleridge	North	0.48	0.46	0.06
AV90	Coleridge	North	0.53	0.45	0.03
AV91	Coleridge	North	0.60	0.38	0.02
**NMW 12.211**	**Coleridge**	**North**	**0.44**	**0.28**	**0.28**
AV2039	Tekapo	Central	0.34	0.64	0.02
**1927.12.18.125**	**Cook**	**Central**	**0.26**	**0.69**	**0.05**
LB2469	Milford	South	0.03	0.04	0.92
